# Supplementary material for: MetaBiome: a multiscale model integrating agent-based and metabolic networks to reveal spatial regulation in gut mucosal microbial communities
Source: mSystems. 2025 Apr 4;10(5):e01652-24. doi: 10.1128/msystems.01652-24 (PMC12090770; doi:10.1128/msystems.01652-24)
Supplement: Supplemental Information — Supplemental figures and tables. [file msystems.01652-24-s0001.pdf]

*Meta*Biome: A Multiscale Model Integrating Agent-Based and Metabolic Networks to Reveal Spatial Regulation in Gut Mucosal Microbial Communities

Javad Aminian-Dehkordi[1,†], Andrew Dickson[1,†], Amin Valiei[1], Mohammad R.K. Mofrad[1,2*]

1. Molecular Cell Biomechanics Laboratory, Departments of Bioengineering and Mechanical Engineering, University of California, Berkeley, California 94720, USA

2. Molecular Biophysics and Integrative Bioimaging Division, Lawrence Berkeley National Lab, Berkeley, California 94720, USA

[†]*These authors contributed equally to this work*

**Corresponding author: mofrad@berkeley.edu*

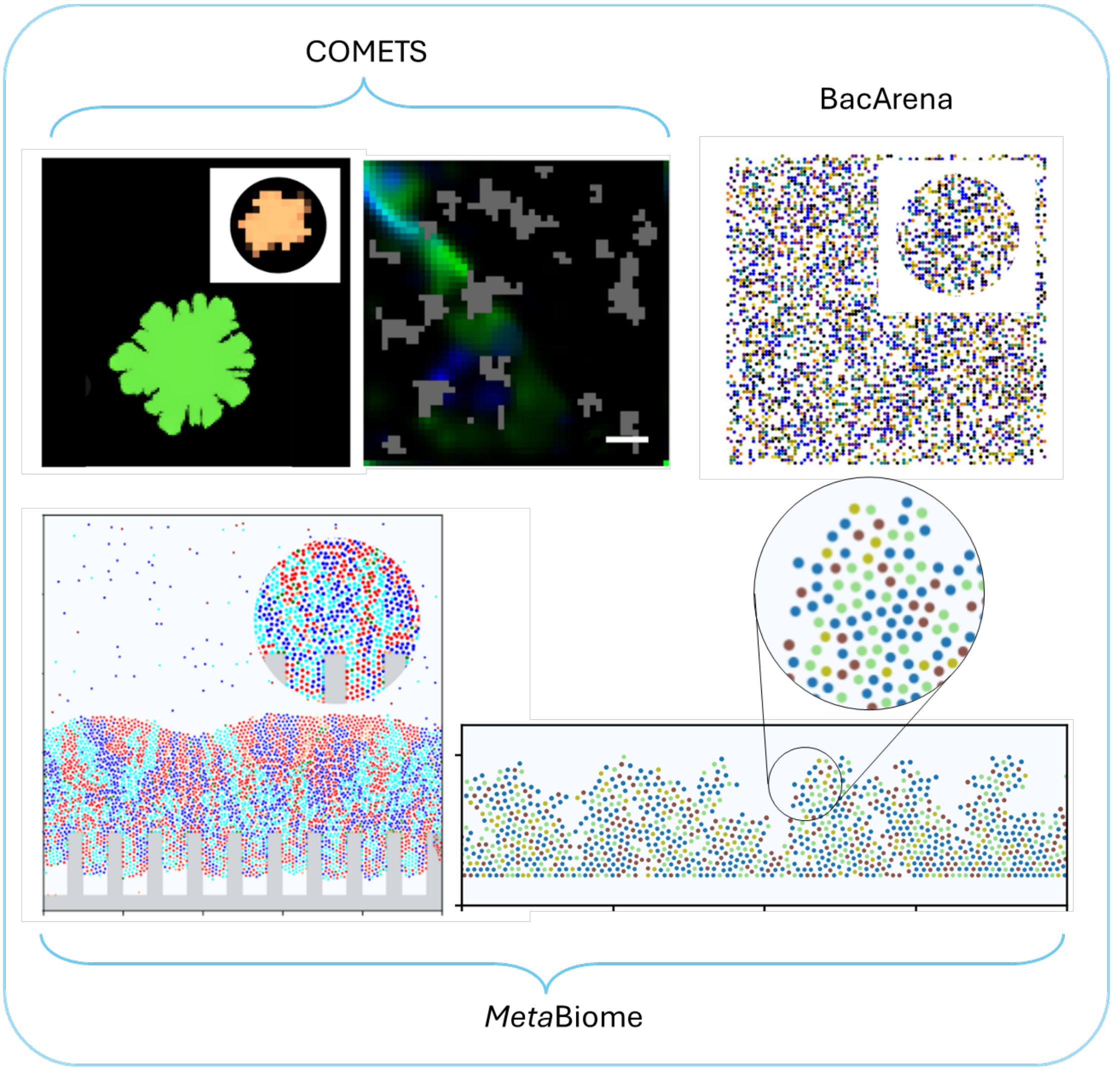

Figure S1- Typical graph outputs from *Meta*Biome and existing frameworks. *Meta*Biome is based on a continuum model of bacterial cells, whereas other platforms are based on the grid-based definition of cells. For more details, see Table S1.

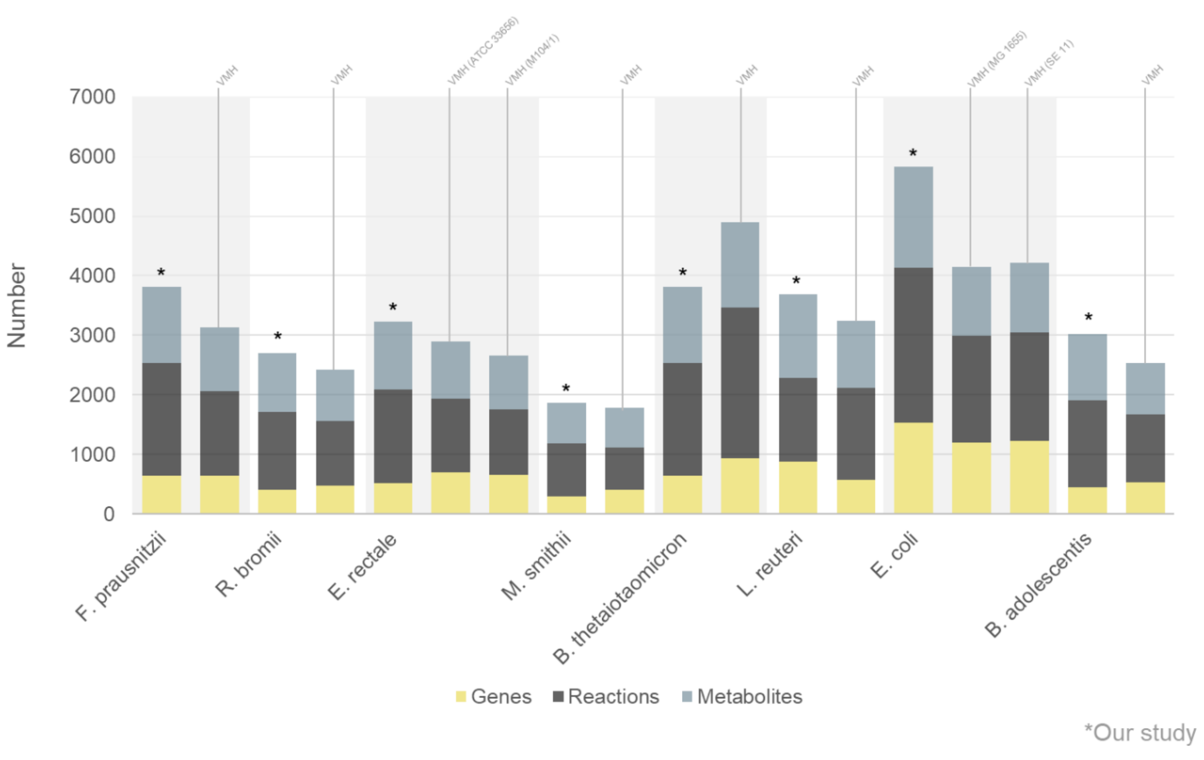

Figure S2- Comparison of reconstructed GEMs in this study with available GEMs on VMH.

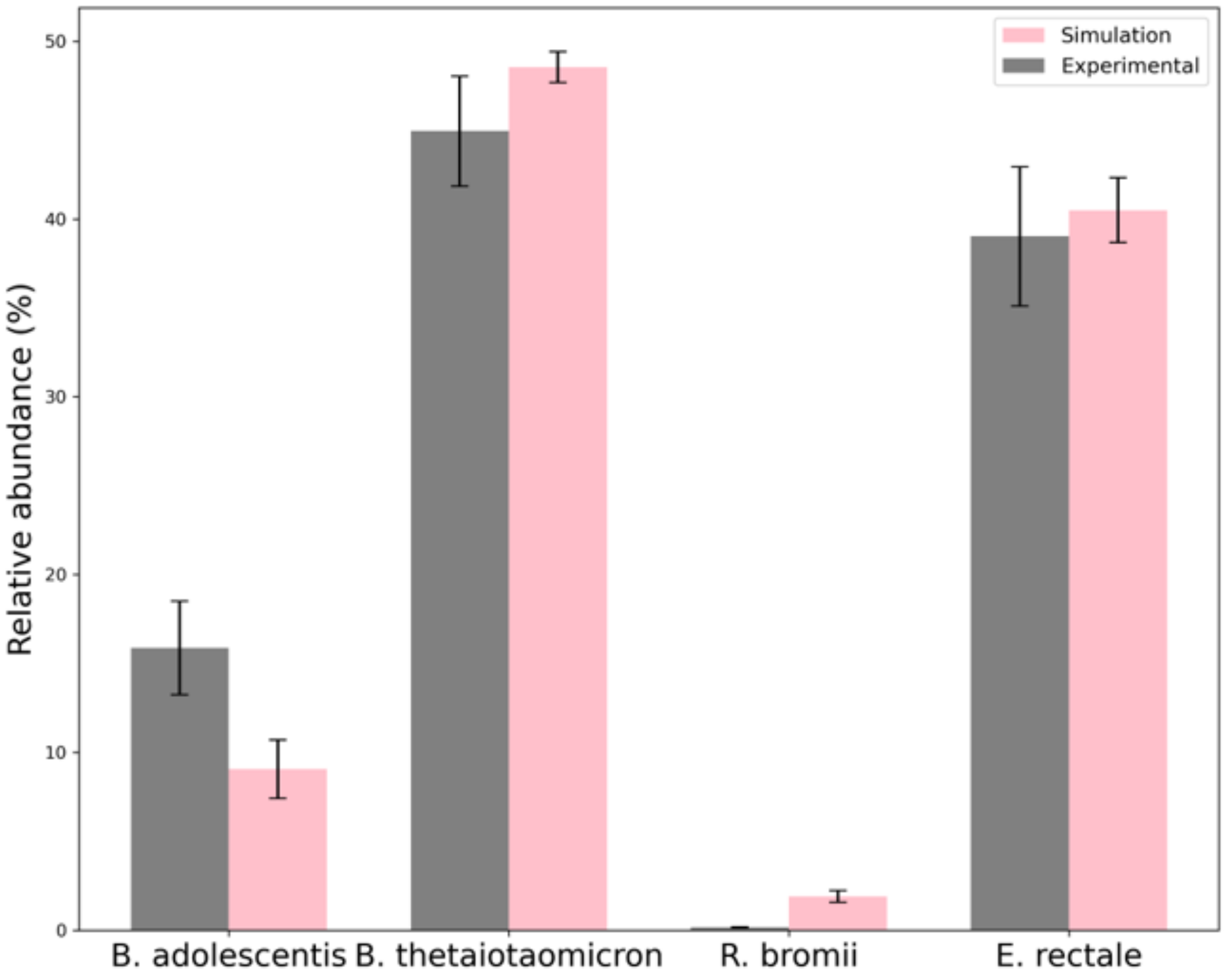

Figure S3- Abundance of bacterial species related to Figure 4.

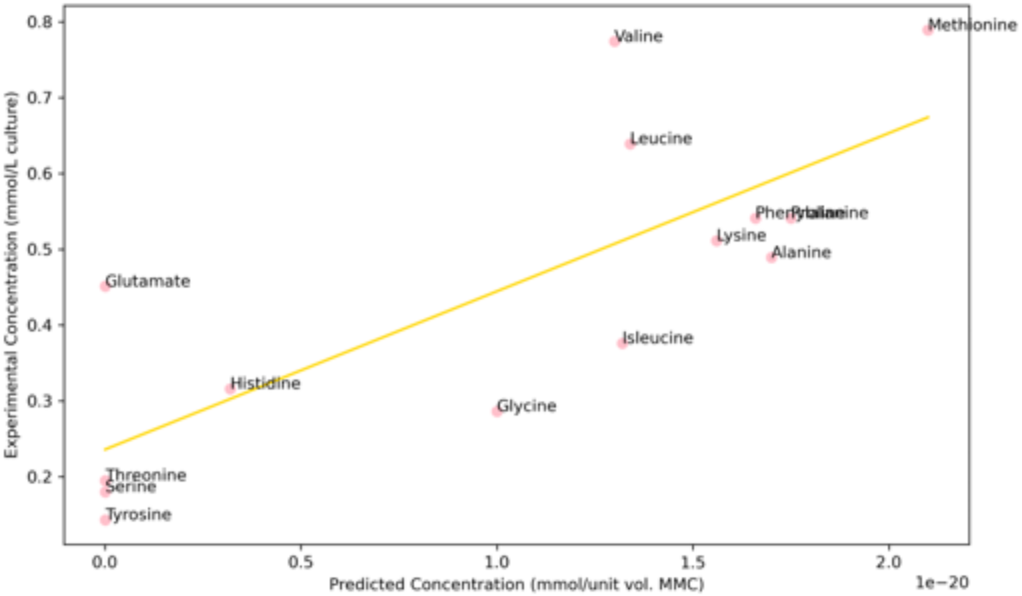

Figure S4- Predicted levels of amino acids in comparison with *in-vitro* experimental data.

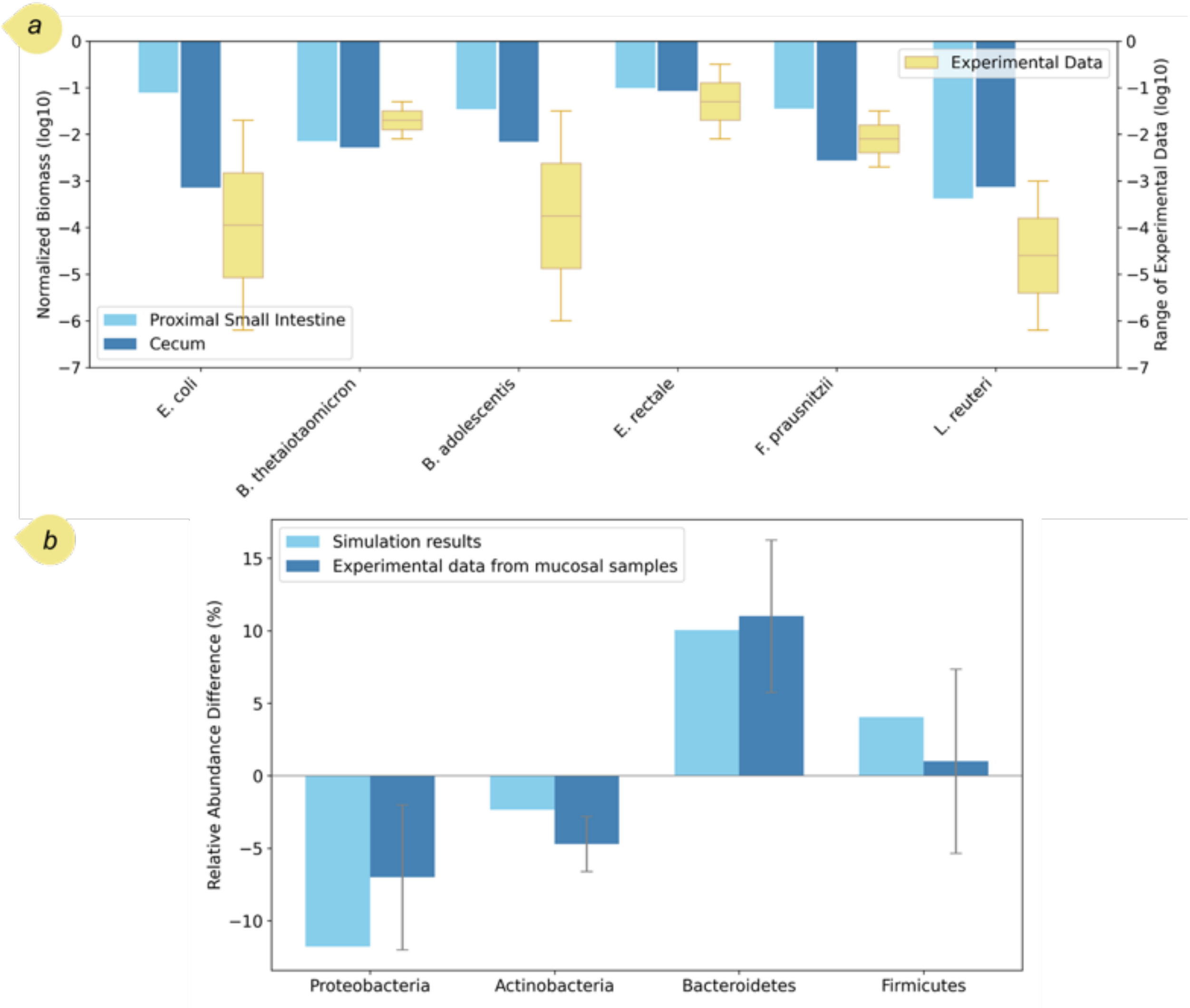

Figure S5- Abundance of bacterial species in MMCs in the proximal small intestine and the cecum upon the averaged diet intervention in overweight individuals, (a) The microbial community was subjected to two different conditions: proximal small intestine and the cecum. Experimental data obtained from human fecal samples by 16S rRNA qCPR are shown in box plots (Shoaie et al. 2015). (b) Changes in the relative abundance of four main phyla in MMCs from the proximal small intestine to the cecum. Simulation results are based on bacterial populations within the domain. Experimental data comprise the gut microbiota in mucosal samples (Friedman et al. 2018).

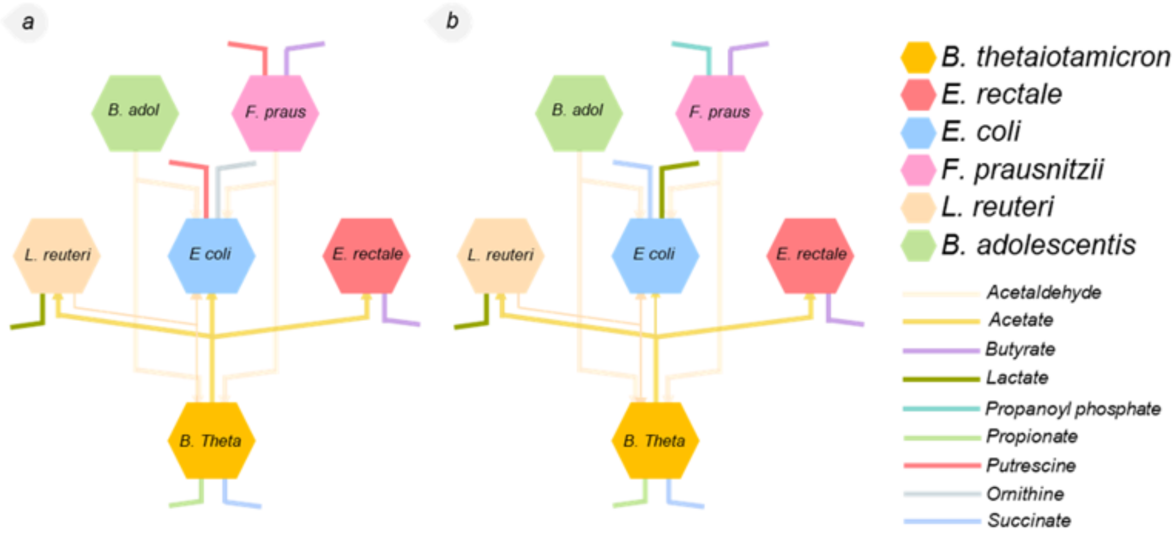

Figure S6- Simplified cross-feeding interactions network related to Figure 5.

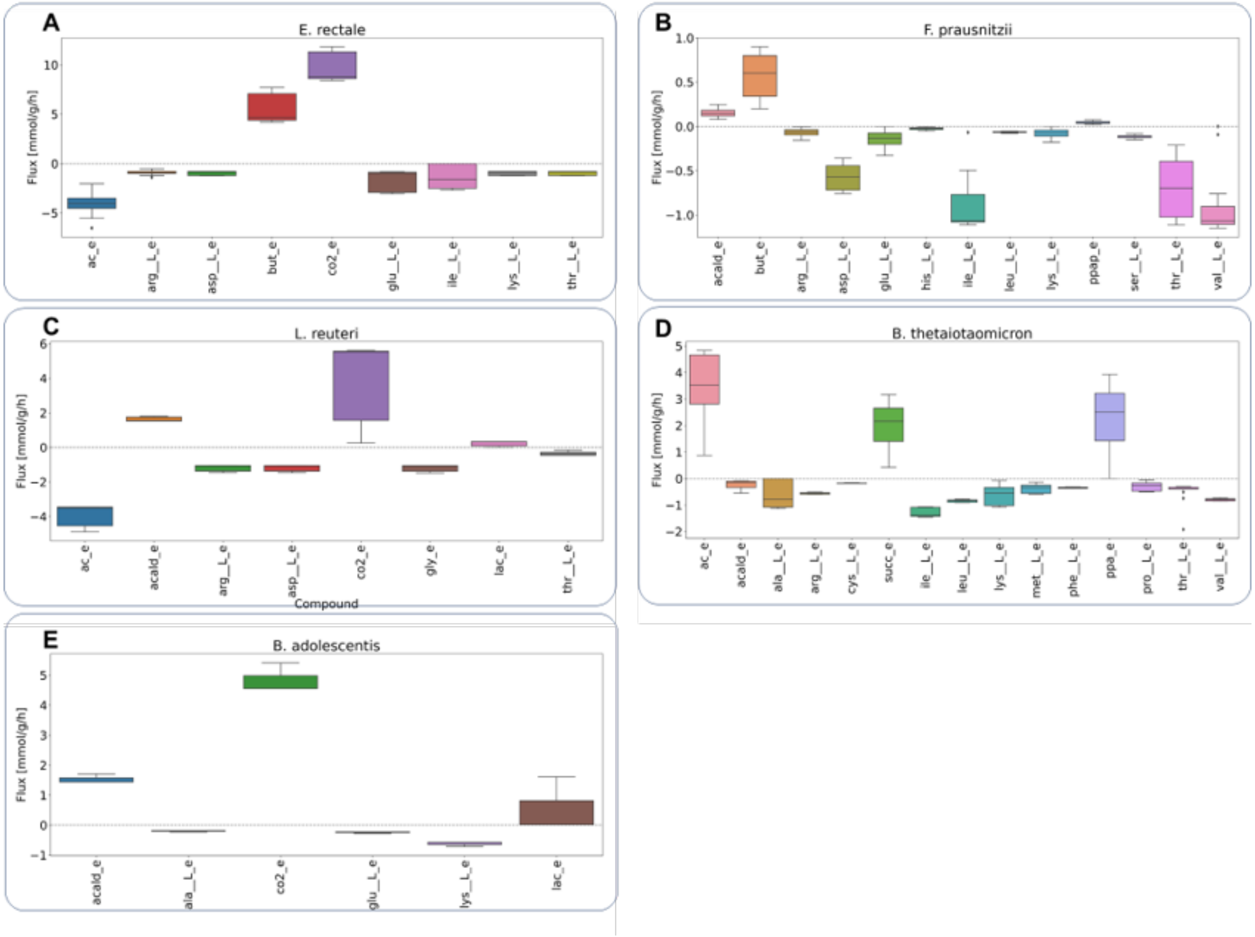

Figure S7- Study of the effect of spatial orientation on microbial metabolism, focusing on metabolite consumption and production across microbiota in the proximal intestine related to Figure 6.

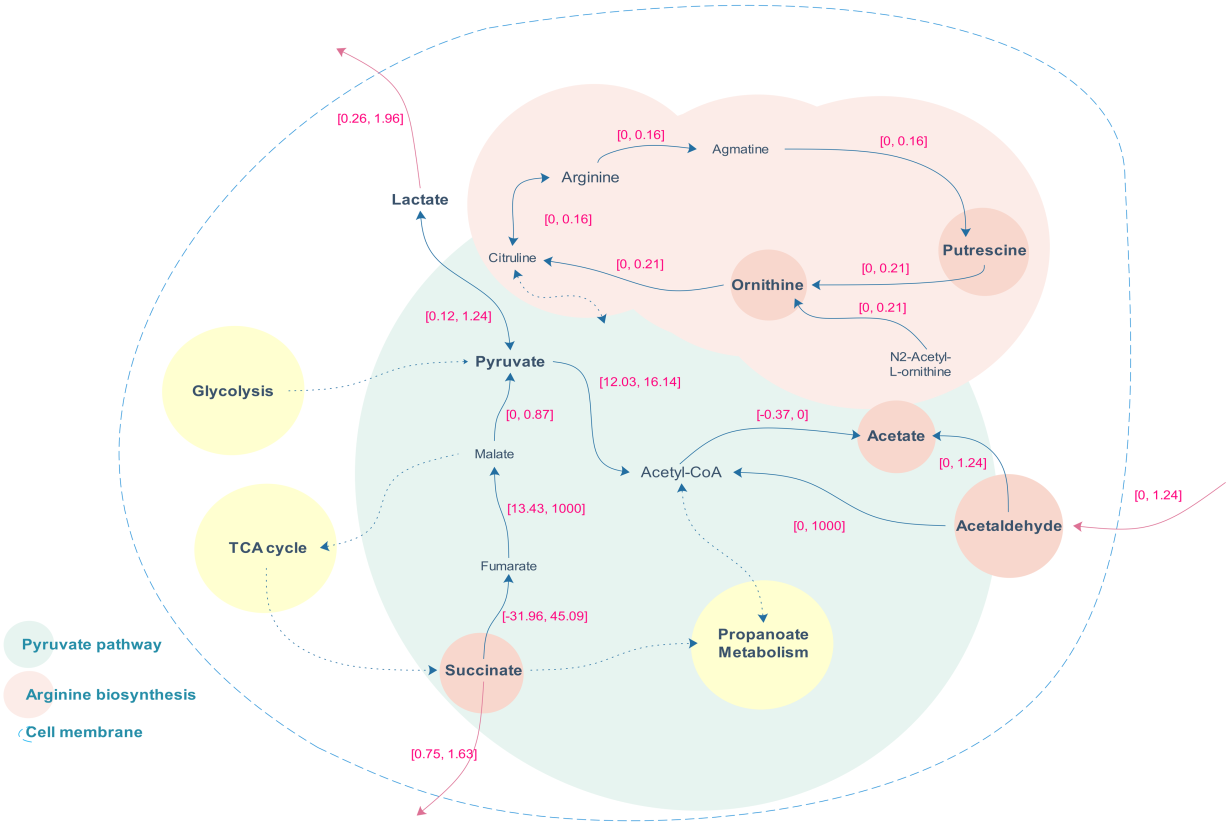

Figure S8- Suboptimal FVA simulations across all *E. coli* agents in the cecum scenario. Values in red correspond to all possible fluxes. FVA simulations for all *E. coli* bacteria in the domain were performed and the union of flux intervals was reported (related to Figure 7).

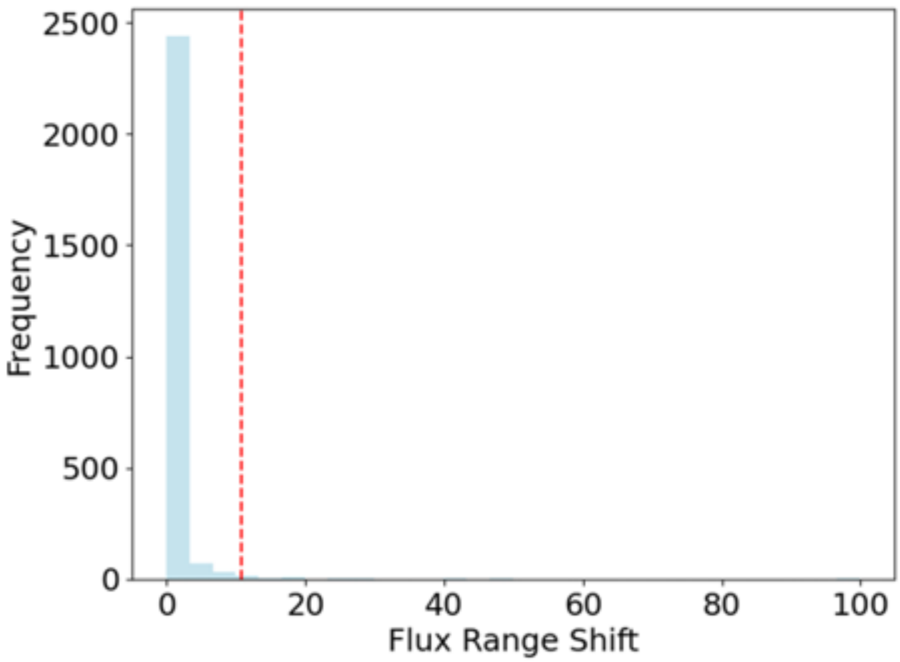

Figure S9- Histogram of maximal flux range shifts obtained from FVA simulations for *E. coli* bacteria, related to Figure 8.

Table S1- Summary of capabilities of different frameworks for studying spatiotemporal features of microbial communities.

**Model**	**Approach**	**Features**
MatNet (Matlab)	cell-as-grid-block design	growth, chemotaxis, one species
BacArena (R)	cell-as-grid-block design	growth, chemotaxis, more than 2 species
COMETS (Matlab/ Python)	population of multiple cells per position (population-based)	growth, more than 2 species, highly applicable to Petri dish colonies
*Meta*Biome (Python)	cell-as-agent design (pure ABM)	growth, chemotaxis, mechanical interactions, varying size, planktonic movement, motility, attachment, no limitation on the microbial composition, specific for biofilms, spatial regulation, pathogen invasion

Table S2- Characteristics and overview of bacteria used in this study.

Phyla	Taxon	NCBI RefSeq assembly	Reactions	Metabolites	GPRs	Transport reactions
Bacteroidetes	*Bacteroides thetaiotaomicron*	GCF_900624795.1	1887	1274	646	288
Bifidobacteria	*Bifidobacterium adolescentis*	GCF_000154085.1	1451	1110	454	276
Firmicutes	*Ruminococcus bromii*	GCF_900291485.1	1318	977	400	275
Firmicutes	*Faecalibacterium prausnitzii*	GCF_032971305.1	1747	1236	504	282
Firmicutes	*Eubacterium rectale*	GCF_000209935.1	1570	1137	522	282
Firmicutes	*Limosilactobacillus reuteri*	GCF_000236455.2	1400	1408	880	145
Euryarchaeota	*Methanobrevibacter smithii*	GCF_000016525.1	890	691	289	103
Proteobacteria	*Escherichia coli*	GCF_000005845.2	2609	1688	1526	390

Table S3- Carbon source utilization assays.

Carbon source		*E. rectale*		*B. thetaiotaomicron*	
		in silico	**Experimenta**	***in silico***	**Experimental**
Arabinose	True-false experiments based on experimental data	-	-	"+"	"+"
Fructose		"+"	"+"	"+"	"+"
Fucose		-	-	-	"+"
Galactose		"+"	"+"	"+"	"+"
D-galacturonic acid		-	-	"+"	"+"
D-glucuronic acid		-	-	"+"	"+"
Glucosamine		"+"	"+"	"+"	"+"
Glucose		"+"	"+"	"+"	"+"
Mannose		-	-	"+"	"+"
L-rhamnose		-	-	-	"+"
Ribose		-	-	"+"	"+"
Xylose		"+"	"+"	"+"	"+"
Cellobiose		"+"	"+"	-	-
Sucrose		-	-	"+"	"+"
Lactose		"+"	"+"	"+"	"+"

Table S4- Checking the production and consumption of amino acids-*in-silico.*

AA	C/P?	*E. rectale*	*B. thetaiotaomicron*	*B. adolescentis*	*F. prausnitzii*	*R. bromii*	*E. coli*
Alanine	Consumption	"+"	"+"	"+"	"+"	"+"	"+"
Glycine		"+"	"+"	"+"	"+"	"+"	"+"
Valine		"+"	"+"	"+"	"+"	"+"	"+"
Leucine		"+"	"+"	"+"	"+"	"+"	"+"
Isoleucine		"+"	"+"	"+"	"+"	"+"	"+"
Threonine		"+"	"+"	"+"	"+"	"+"	"+"
Serine		"+"	"+"	"+"	"+"	"+"	"+"
Proline		"+"	"+"	"+"	"+"	"+"	"+"
Glutamate		"+"	"+"	"+"	"+"	"+"	"+"
Methionine		"+"	"+"	"+"	"+"	"+"	"+"
Cysteine		"+"	"+"	"+"	"+"	"+"	"+"
Phenylalanine		"+"	"+"	"+"	"+"	"+"	"+"
Lysine		"+"	"+"	"+"	"+"	"+"	"+"
Histidine		"+"	"+"	"+"	"+"	"+"	"+"
Tyrosine		"+"	"+"	"+"	"+"	"+"	"+"
	**C/P?**	***E. rectale***	***B. thetaiotaomicron***	***B. adolescentis***	***F. prausnitzii***	***R. bromii***	***E. coli***
Alanine	Production	"+"	"+"	"+"	"+"	"+"	"+"
Glycine		"+"	"+"	"+"	"+"	"+"	"+"
Valine		"+"	"+"	"+"	"+"	"+"	"+"
Leucine		"+"	"+"	"+"	"+"	"+"	"+"
Isoleucine		"+"	"+"	"+"	"+"	"+"	"+"
Threonine		"+"	"+"	"+"	"+"	"+"	"+"
Serine		"+"	"+"	"+"	"+"	"+"	"+"
Proline		"+"	"+"	"+"	"+"	"+"	"+"
Glutamate		"+"	"+"	"+"	"+"	"+"	"+"
Methionine		"+"	"+"	"+"	"+"	"+"	"+"
Cysteine		"+"	"+"	"+"	"+"	"+"	"+"
Phenylalanine		"+"	"+"	"+"	"+"	"+"	"+"
Lysine		"+"	"+"	"+"	"+"	"+"	"+"
Histidine		"+"	"+"	"+"	"+"	"+"	"+"
Tyrosine		"+"	"+"	"+"	"+"	"+"	"+"

Ref: Shoaeie *et al.* (2015)

Table S5- Media defined for different scenarios.

Scenario	Medium	Constraint-based model	Oxygen gradients
1- Mouse model cases for butyrate production	M2 + Glucans	FBA	✗
2- *in-vitro* microbial community	M2 + Starch + cellobiose	FBA	✗
3-High-protein diet	High-protein diet	*p*FBA	✓

Table S6- Details of Medium used for Scenario 1.

Components	Medium (mmol/lit)	Initial condition
Glucans	7.75	7.75
Acetate	0	1.57
M2	-	-

Metabolites included in the environment layer
Acetate, Butyrate, Propionate, Glucose

Table S7- Details of Medium used for Scenario 1 with *M. smithii.*

Components	Medium (mmol/lit)	Initial condition
Glucans	7.75	7.75
Acetate	0	1.57
M2	-	-

Metabolites included in the environment layer
Acetate, Butyrate, Propionate, Succinate, Starch, Cellobiose

Table S8- Details of Medium used for Scenario 2.

Components	Medium (mmol/lit)	Initial condition
Cellobiose	5.84	0
Starch	6.17	0
M2	-	-

Metabolites included in the environment layer
Acetate, Butyrate, Propionate, Succinate, Glucose, CH4, Amino acids

Table S9- Details of high-protein diet: foods averaged over overweight individuals

Components	Amount in gr
breadandsimilarproduct	28.8202222
feculents	34.8248889
cereals	19.666
legumineuses	8.598
tubercules	6.11666667
cereals_breakfast	0
milk	20.2168889
yogourt_dairy	139.741111
cheese	59.6317778
meatand derivate	64.4982222
viande de boucherie	56.3397778
whitemeat	29.7313333
redmeat	26.6093333
sausageand derivate	14.428
seaproducts	29.9831111
fish	20.426
seafish	21.2066667
riverfish	0.31755556
mollusques	2.92266667
crustaces	6.09511111
eggs	4.98355556
fruitsandvegetable	525.650444
fruits	207.118
rawfruits	200.578444
cookedfruits	5.55555556
vegetable	318.532667
rawvegetable	140.278444

Metabolites included in the environment layer
Listed in Table S10

cookedvegetable	177.777556
origine vegetale	11.3355556
viennoiseries patisseries biscuits	4.09111111
Soupes	45.9517778
boissons	1116.62422
eau	790.783333
boissons non sucrees sans alcool	254.762222
boissons sucrees sans alcool	65.1851111
boissons alcoolisees	8.53733333
dairy,products	219.590222

Table S10- Nutrient content table in mass fractions*

Nutrients	Bread and bread products	Cereals e.g. rice, pasta	Potatoes, mashed, dehydrated, granules without milk, dry form	Breakfast cereals	Beans, kidney, mature seeds	Milk	Yogurt, plain, whole milk	Cheese, brie	Delicatessen meats e.g. ham	Beef, chuck eye roast, boneless, America's Beef Roast	White meat e.g. chicken	Fish and fish products	Eggs and egg dishes	Vegetables, mixed, frozen, unprepared	Fruits
Tryptophan	0.0013688	0.00132705	0.000804	0.002537	0.005552	0.002266	0.001525	0.013854	0.009013	0.011806	0.012538	0.010709	0.012297	0.001674	0.000555
Threonine	0.00444861	0.00348351	0.002769	0.006989	0.022543	0.007534	0.010824	0.032311	0.042408	0.046602	0.028315	0.051657	0.04094	0.006549	0.001666
Isoleucine	0.0038386	0.0043461	0.002789	0.007584	0.023765	0.009168	0.014407	0.043669	0.040444	0.045044	0.051025	0.04623	0.049407	0.007977	0.001666
Leucine	0.00685889	0.00842678	0.004358	0.01359	0.038534	0.016807	0.026679	0.082993	0.072683	0.085178	0.097816	0.075693	0.079965	0.010882	0.002776
Lysine	0.00319883	0.00275363	0.004665	0.008322	0.030539	0.014858	0.023706	0.079637	0.078807	0.092581	0.116416	0.085239	0.067153	0.009749	0.002221
Methionine	0.00156222	0.00235552	0.0013	0.003313	0.005552	0.004636	0.007775	0.02547	0.026346	0.030003	0.034584	0.027961	0.02798	0.00197	0.000555
Cystine	0.00229126	0.0019574	0.001122	0.00374	0.006108	0.001054	0.002439	0.004905	0.010978	0.010949	0.012455	0.007705	0.020028	0.001477	0.000555
Phenylalanine	0.00468666	0.00510915	0.005062	0.009539	0.026985	0.009168	0.014407	0.049821	0.037035	0.040134	0.047829	0.040948	0.05007	0.006844	0.001666
Tyrosine	0.00245492	0.00278681	0.002313	0.005643	0.018434	0.008957	0.013339	0.051628	0.030968	0.03651	0.03965	0.035957	0.036743	0.004235	0.00111
Valine	0.00461227	0.00603809	0.004377	0.009746	0.02754	0.011591	0.021877	0.057652	0.043159	0.047615	0.053101	0.053305	0.063176	0.008568	0.002221
Arginine	0.00462715	0.00836043	0.004427	0.017693	0.029206	0.005058	0.007927	0.031622	0.059106	0.069202	0.074359	0.062367	0.060378	0.011079	0.002221
Histidine	0.00223174	0.00238869	0.001548	0.005048	0.014992	0.005321	0.006555	0.030805	0.034897	0.033939	0.042639	0.026313	0.022752	0.004185	0.00111
Alanine	0.00364518	0.00557362	0.002928	0.010095	0.02221	0.006006	0.011281	0.036957	0.054772	0.0595	0.057627	0.063384	0.05412	0.007041	0.001666
Aspartic acid	0.00517765	0.0102183	0.019088	0.018857	0.069739	0.015174	0.020962	0.058082	0.086665	0.094763	0.068671	0.124782	0.097857	0.017381	0.003887
Glutamic acid	0.03064929	0.01794838	0.014562	0.0413	0.065408	0.039831	0.051757	0.188745	0.135082	0.167589	0.131529	0.140676	0.123187	0.022404	0.005552
Glycine	0.00389811	0.00461151	0.002491	0.011208	0.018434	0.00353	0.006403	0.01708	0.051017	0.045823	0.049282	0.061204	0.031809	0.005958	0.00111
Proline	0.010147	0.00620397	0.002809	0.010276	0.021655	0.017492	0.031329	0.105795	0.041195	0.042355	0.043594	0.042014	0.0377	0.004038	0.001666
Serine	0.00461227	0.00471103	0.003315	0.00884	0.028651	0.010695	0.016388	0.050252	0.039115	0.040407	0.03857	0.043855	0.071423	0.00778	0.001666
Carbohydrate, by difference	0.82976254	0.87154137	0.848793	0.689195	0.524153	0.810854	0.71042	0.038721	0.106309	0	0	0	0.053015	0.663253	0.832871
Fiber, total dietary	0.06992799	0.02985867	0.070476	0.116484	0	0	0	0	0	0	0	0	0	0.196957	0.133259

*Molecular weights of compounds are given in Table S12.

Table S10 – Continued.

Nutrients	Fats and oils	Sweets, confectionary and table sugar	Cake, shortcake, biscuit-type	Soup, tomato bisque, canned, condensed	Savoury snacks, pies and pizzas	Nuts and seeds	Drinks without sugar without alcohol e.g. tea, coffee	Drinks with sugar without alcohol e.g. soda, fruit juice	Drinks with alcohol e.g. wine, beer	Tubercules	Féculents	Légumineuses	Mollusques	Crustacés
Tryptophan	0.012632	0	0.001392	0.000861	0.002537	0.004891	0	0	0	0.000955	0.001341	0.001775	0.004318	0.008495
Threonine	0.04	0	0.00337	0.002489	0.008612	0.01106	0	0	0	0.003819	0.003577	0.005324	0.025907	0.042476
Isoleucine	0.053684	0	0.004358	0.002872	0.011085	0.013783	0	0	0	0.004773	0.004135	0.006507	0.030225	0.048544
Leucine	0.087368	0	0.008222	0.00469	0.02202	0.025399	0	0	0	0.00716	0.00693	0.009465	0.043178	0.072816
Lysine	0.070526	0	0.003608	0.00335	0.015753	0.01319	0	0	0	0.005728	0.002794	0.008282	0.047496	0.078883
Methionine	0.022105	0	0.002106	0.001101	0.005606	0.004224	0	0	0	0.001909	0.002235	0.001775	0.015544	0.03034
Cystine	0.008421	0	0.002106	0.000909	0.003624	0.005317	0	0	0	0.001432	0.002794	0.00142	0.004318	0.008495
Phenylalanine	0.043158	0	0.00553	0.002872	0.012875	0.017655	0	0	0	0.004773	0.004694	0.006507	0.021589	0.036408
Tyrosine	0.043158	0	0.003717	0.002201	0.009955	0.012523	0	0	0	0.003341	0.003912	0.003549	0.017271	0.03034
Valine	0.06	0	0.004999	0.003254	0.013813	0.017303	0	0	0	0.005728	0.004471	0.007099	0.021589	0.036408
Arginine	0.032632	0	0.004395	0.002441	0.011106	0.041535	0	0	0	0.003819	0.004471	0.008874	0.03886	0.06068
Histidine	0.024211	0	0.002564	0.001627	0.007439	0.008892	0	0	0	0.002387	0.003353	0.004733	0.025907	0.042476
Alanine	0.030526	0	0.003608	0.002393	0.010104	0.014932	0	0	0.000908	0.004773	0.003912	0.005916	0.051813	0.084951
Aspartic acid	0.067368	0	0.005347	0.00737	0.017629	0.038089	0	0	0.00121	0.005728	0.005588	0.009465	0.060449	0.097087
Glutamic acid	0.187368	0	0.034739	0.028954	0.068916	0.081939	0	0	0.003554	0.01432	0.016765	0.014198	0.07772	0.121359
Glycine	0.018947	0	0.003681	0.001914	0.009294	0.019711	0	0	0.000983	0.003341	0.003353	0.004733	0.034542	0.06068
Proline	0.086316	0	0.012471	0.005169	0.025345	0.01621	0	0	0.002647	0.004773	0.004471	0.005916	0.017271	0.03034
Serine	0.048421	0	0.005622	0.003302	0.013557	0.016988	0	0	0	0.004773	0.004694	0.007099	0.030225	0.048544
Carbohydrate, by difference	0.063158	1	0.888165	0.883944	0.681702	0.469627	1	0.991266	0.990698	0.811456	0.804739	0.709891	0.431779	0.06068
Fiber, total dietary	0	0	0	0.038287	0.049028	0.166732	0	0.008734	0	0.105012	0.111769	0.177473	0	0

Table S11- List of common metabolites present in GEMs of species related to overweight individuals' MMCs.

ID	Name	Formula	Charge	Compartment
asp__L_e	L-Aspartate	C4H6NO4	-1	C_e
sucr_e	Sucrose C12H22O11	C12H22O11	0	C_e
cm_e	Chloramphenicol	C11H12Cl2N2O5	0	C_e
xylan4_e	Xylan (4 backbone units, 1 glcur side chain)	C26H41O23	0	C_e
starch_e	Starch C12H20O10	C12H20O10	0	C_e
glyb_e	Glycine betaine	C5H11NO2	0	C_e
ascb__L_e	L-Ascorbate	C6H8O6	0	C_e
chols_e	Choline sulfate	C5H13NO4S	0	C_e
thymd_e	Thymidine C10H14N2O5	C10H14N2O5	0	C_e
gcald_e	Glycolaldehyde	C2H4O2	0	C_e
ac_e	Acetate	C2H3O2	-1	C_e
cellb_e	Cellobiose	C12H22O11	0	C_e
gsn_e	Guanosine	C10H13N5O5	0	C_e
fald_e	Formaldehyde	CH2O	0	C_e
5drib_e	5'-deoxyribose	C5H10O4	0	C_e
5mdru1p_e	5-Methylthio-5-deoxy-D-ribulose 1-phosphate	C6H11O7PS	-2	C_e
12ppd__R_e	(R)-Propane-1,2-diol	C3H8O2	0	C_e
gua_e	Guanine	C5H5N5O	0	C_e
25dkglcn_e	2,5-diketo-D-gluconate	C6H7O7	-1	C_e
inost_e	Myo-Inositol	C6H12O6	0	C_e
skm_e	Shikimate	C7H9O5	-1	C_e
glyc2p_e	Glycerol 2-phosphate	C3H7O6P	-2	C_e
galur_e	D-Galacturonate	C6H9O7	-1	C_e
ptrc_e	Putrescine	C4H14N2	2	C_e
xylb_e	Xylobiose	C10H18O9	0	C_e
ura_e	Uracil	C4H4N2O2	0	C_e
p_xyl_e	P-methyltoluene	C8H10	0	C_e
rib__D_e	D-Ribose	C5H10O5	0	C_e
3ump_e	3 UMP C9H11N2O9P	C9H11N2O9P	-2	C_e
enter_e	Enterochelin	C30H26N3O15	-1	C_e
gmp_e	GMP C10H12N5O8P	C10H12N5O8P	-2	C_e
for_e	Formate	CHO2	-1	C_e
pydam_e	Pyridoxamine	C8H13N2O2	1	C_e

ala__L_e	L-Alanine	C3H7NO2	0	C_e
m_xyl_e	M-Xylene	C8H10	0	C_e
pro__L_e	L-Proline	C5H9NO2	0	C_e
fru_e	D-Fructose	C6H12O6	0	C_e
phe__L_e	L-Phenylalanine	C9H11NO2	0	C_e
ade_e	Adenine	C5H5N5	0	C_e
but_e	Butyrate (n-C4:0)	C4H7O2	-1	C_e
gal_bD_e	Beta D-Galactose	C6H12O6	0	C_e
acgam_e	N-Acetyl-D-glucosamine	C8H15NO6	0	C_e
metox__R_e	L methionine R oxide C5H11NO3S	C5H11NO3S	0	C_e
thm_e	Thiamin	C12H17N4OS	1	C_e
coa_e	Coenzyme A	C21H32N7O16P3S	-4	C_e
chtbs_e	N, N'-diacetylchitobiose	C16H28N2O11	0	C_e
glcn_e	D-Gluconate	C6H11O7	-1	C_e
arbt_e	Arbutin C12H16O7	C12H16O7	0	C_e
crn_e	L-Carnitine	C7H15NO3	0	C_e
malttr_e	Maltotriose C18H32O16	C18H32O16	0	C_e
spmd_e	Spermidine	C7H22N3	3	C_e
dcyt_e	Deoxycytidine	C9H13N3O4	0	C_e
malthx_e	Maltohexaose	C36H62O31	0	C_e
d23hb_e	2,3-dihydroxybenzoate	C7H6O4	0	C_e
melib_e	Melibiose C12H22O11	C12H22O11	0	C_e
malthp_e	Maltoheptaose	C42H72O36	0	C_e
15dap_e	1,5-Diaminopentane	C5H16N2	2	C_e
stfrnB_e	Staphyloferrin B	C19H22N2O18	0	C_e
4ahmmp_e	4-Amino-5-hydroxymethyl-2-methylpyrimidine	C6H9N3O	0	C_e
lac__D_e	D-Lactate	C3H5O3	-1	C_e
lcts_e	Lactose C12H22O11	C12H22O11	0	C_e
trp__L_e	L-Tryptophan	C11H12N2O2	0	C_e
cit_e	Citrate	C6H5O7	-3	C_e
gly_e	Glycine	C2H5NO2	0	C_e
chol_e	Choline C5H14NO	C5H14NO	1	C_e
stfrnA_e	Staphyloferrin A	C17H24N2O14	0	C_e
indole_e	Indole	C8H7N	0	C_e
2mba_e	2 methyl butanoic acid	C5H9O2	0	C_e
ppap_e	Propanoyl phosphate	C3H5O5P	-2	C_e
ribflv_e	Riboflavin C17H20N4O6	C17H22N4O6	0	C_e
dtmp_e	DTMP C10H13N2O8P	C10H13N2O8P	-2	C_e

cytd_e	Cytidine	C9H13N3O5	0	C_e
gly_asn__L_e	Gly asn L C6H11N3O4	C6H11N3O4	0	C_e
2pglyc_e	2-Phosphoglycolate	C2H2O6P	-3	C_e
val__L_e	L-Valine	C5H11NO2	0	C_e
R3hdec4e_e	3 Hydroxy 4Z decenic acid	C10H17O3	0	C_e
dextrin_e	Dextrin C12H20O10	C12H20O10	0	C_e
dca_e	Decanoate (n-C10:0)	C10H19O2	-1	C_e
adn_e	Adenosine	C10H13N5O4	0	C_e
leu__L_e	L-Leucine	C6H13NO2	0	C_e
ttrcyc_e	Tetracycline	C22H24N2O8	0	C_e
R_3hdcaa_e	3 hydroxydecanoic acid	C10H19O3	0	C_e
dad_2_e	Deoxyadenosine	C10H13N5O3	0	C_e
malt_e	Maltose C12H22O11	C12H22O11	0	C_e
acald_e	Acetaldehyde	C2H4O	0	C_e
din_e	Deoxyinosine	C10H12N4O4	0	C_e
metox_e	L Methionine S oxide C5H11NO3S	C5H11NO3S	0	C_e
xan_e	Xanthine	C5H4N4O2	0	C_e
2mpa_e	2 methylpropanoic acid	C4H7O2	0	C_e
udpg_e	UDPglucose	C15H22N2O17P2	-2	C_e
fum_e	Fumarate	C4H2O4	-2	C_e
pheme_e	Protoheme C34H30FeN4O4	C34H30FeN4O4	-2	C_e
gal_e	D-Galactose	C6H12O6	0	C_e
cys__L_e	L-Cysteine	C3H7NO2S	0	C_e
ala_B_e	Beta-Alanine	C3H7NO2	0	C_e
apc_e	Ampicillin	C16H19N3O4S	0	C_e
sbt__D_e	D-Sorbitol	C6H14O6	0	C_e
orn_e	Ornithine	C5H13N2O2	1	C_e
anhgm_e	N-Acetyl-D-glucosamine(anhydrous)N-Acetylmuramic acid	C19H29N2O12	-1	C_e
agm_e	Agmatine	C5H16N4	2	C_e
arab__L_e	L-Arabinose	C5H10O5	0	C_e
cyan_e	Hydrogen cyanide	CHN	0	C_e
glu__L_e	L-Glutamate	C5H8NO4	-1	C_e
glcr_e	D-Glucarate	C6H8O8	-2	C_e
2hxmp_e	2 Hydroxymethyl phenol C7H8O2	C7H8O2	0	C_e
btn_e	Biotin	C10H15N2O3S	-1	C_e
xyl__D_e	D-Xylose	C5H10O5	0	C_e
thr__L_e	L-Threonine	C4H9NO3	0	C_e
cmp_e	CMP C9H12N3O8P	C9H12N3O8P	-2	C_e

mnl_e	D-Mannitol	C6H14O6	0	C_e
thym_e	Thymine C5H6N2O2	C5H6N2O2	0	C_e
uri_e	Uridine	C9H12N2O6	0	C_e
fol_e	Folate	C19H17N7O6	-2	C_e
ad_e	Acetamide	C2H5NO	0	C_e
tartr__D_e	D-tartrate	C4H4O6	-2	C_e
tol_e	Toluene	C7H8	0	C_e
salchs4fe_e	Salmochelin-S4-Fe-III	C42FeH46N3O25	0	C_e
xyl3_e	Xylotriose	C15H26O13	0	C_e
tyr__L_e	L-Tyrosine	C9H11NO3	0	C_e
r5p_e	Alpha-D-Ribose 5-phosphate	C5H9O8P	0	C_e
urea_e	Urea CH4N2O	CH4N2O	0	C_e
pnto__R_e	(R)-Pantothenate	C9H16NO5	-1	C_e
lipoate_e	Lipoate	C8H13O2S2	-1	C_e
meoh_e	Methanol	CH4O	0	C_e
glucan4_e	Beta-1,3/1,4-glucan (Barley, n=4, Glc beta1->3,4 Glc)	C24H42O21	0	C_e
14glucan_e	1,4-alpha-D-glucan	C36H62O31	0	C_e
istfrnA_e	Iron bound extracellular staphyloferrin A	C17FeH24N2O14	0	C_e
lmn2_e	Laminaribiose	C12H22O11	0	C_e
progly_e	L-Prolinylglycine	C7H12N2O3	0	C_e
lyx__L_e	L-Lyxose	C5H10O5	0	C_e
etoh_e	Ethanol	C2H6O	0	C_e
g3ps_e	Glycerophosphoserine	C6H13NO8P	-1	C_e
ch4_e	Methane	CH4		C_e
5mcsn_e	5-Methylcytosine	C5H7N3O	0	C_e
novbcn_e	Novobiocin	C31H35N2O11	-1	C_e
fe3pyovd_kt_e	Ferrypyoverdine P putida KT2440 specific	C27FeH48N10O15	0	C_e
4hpro_LT_e	Trans 4 Hydroxy L proline C5H9NO3	C5H9NO3	0	C_e
tartr__L_e	L-tartrate	C4H4O6	-2	C_e
arg__L_e	L-Arginine	C6H15N4O2	1	C_e
acnam_e	N-Acetylneuraminate	C11H18NO9	-1	C_e
actn__R_e	R Acetoin C4H8O2	C4H8O2	0	C_e
glyc3p_e	Glycerol 3-phosphate	C3H7O6P	-2	C_e
lys__L_e	L-Lysine	C6H15N2O2	1	C_e
glucan6_e	Beta-1,3/1,4-glucan (Barley, n=6, Glc beta1->3,4 Glc)	C36H62O31	0	C_e
fusa_e	Fusidic acid	C31H47O6	-1	C_e
udcpp_e	Undecaprenyl phosphate	C55H90O4P	-1	C_e

3gmp_e	Guanosine 3 phosphate C10H12N5O8P	C10H12N5O8P	-2	C_e
raffin_e	Raffinose C18H32O16	C18H32O16	0	C_e
thrp_e	L-Threonine O-3-phosphate	C4H8NO6P	-2	C_e
2ddglcn_e	2-Dehydro-3-deoxy-D-gluconate	C6H9O6	-1	C_e
ins_e	Inosine	C10H12N4O5	0	C_e
met_L_ala__L_e	Met L ala L C8H16N2O3S	C8H15N2O3S	0	C_e
carn_e	L-Carnosine	C9H14N4O3	0	C_e
acglu_e	N-Acetyl-L-glutamate	C7H9NO5	-2	C_e
his__L_e	L-Histidine	C6H9N3O2	0	C_e
mmet_e	S-Methyl-L-methionine	C6H14NO2S	0	C_e
ser__D_e	D-Serine	C3H7NO3	0	C_e
ser__L_e	L-Serine	C3H7NO3	0	C_e
nac_e	Nicotinate	C6H4NO2	-1	C_e
g3pg_e	Glycerophosphoglycerol	C6H14O8P	0	C_e
succ_e	Succinate	C4H4O4	-2	C_e
metglcur_e	1-O-methyl-Beta-D-glucuronate	C7H11O7	0	C_e
alltn_e	Allantoin	C4H6N4O3	0	C_e
gln__L_e	L-Glutamine	C5H10N2O3	0	C_e
4hphac_e	4-Hydroxyphenylacetate	C8H7O3	-1	C_e
26dap__M_e	Meso-2,6-Diaminoheptanedioate	C7H14N2O4	0	C_e
cgly_e	Cys Gly C5H10N2O3S	C5H10N2O3S	0	C_e
drib_e	Deoxyribose C5H10O4	C5H10O4	0	C_e
csn_e	Cytosine	C4H5N3O	0	C_e
feenter_e	Fe-enterobactin	C30FeH26N3O15	0	C_e
tcynt_e	Thiocyanate	CNS	-1	C_e
met__D_e	D-Methionine	C5H11NO2S	0	C_e
nmn_e	NMN C11H14N2O8P	C11H14N2O8P	-1	C_e
Larab_e	Alpha L Arabinan C15H24O12	C15H24O12	0	C_e
tre_e	Trehalose	C12H22O11	0	C_e
arbt6p_e	Arbutin 6-phosphate	C12H15O10P	-2	C_e
hqn_e	Hydroquinone	C6H6O2	0	C_e
taur_e	Taurine C2H7NO3S	C2H7NO3S	0	C_e
mal__L_e	L-Malate	C4H4O5	-2	C_e
LalaLglu_e	L-alanine-L-glutamate	C8H13N2O5	-1	C_e
3cmp_e	3 CMP C9H12N3O8P	C9H12N3O8P	-2	C_e
peamn_e	Phenethylamine	C8H12N	1	C_e
bz_e	Benzoate	C7H5O2	-1	C_e
ppa_e	Propionate (n-C3:0)	C3H5O2	-1	C_e

istfrnB_e	Iron bound extracellular staphyloferrin B	C19FeH22N2O18	0	C_e
3amp_e	3 AMP C10H12N5O7P	C10H12N5O7P	-2	C_e
gam_e	D-Glucosamine	C6H14NO5	1	C_e
4abut_e	4-Aminobutanoate	C4H9NO2	0	C_e
salchs4_e	Salmochelin-S4	C42H46N3O25	0	C_e
4oxptn_e	4 Oxopentanoate	C5H7O3	0	C_e
hxan_e	Hypoxanthine	C5H4N4O	0	C_e
gthrd_e	Reduced glutathione	C10H16N3O6S	-1	C_e
fe3dhbzs3_e	2-3-dihydroxybenzoylserine trimer-Fe-III	C30FeH28N3O16	0	C_e
pser__L_e	O-Phospho-L-serine	C3H6NO6P	-2	C_e
salcn_e	Salicin C13H18O7	C13H18O7	0	C_e
dhptd_e	4,5-dihydroxy-2,3-pentanedione	C5H8O4	0	C_e
glc__aD_e	Alpha D-glucose	C6H12O6	0	C_e
g3pc_e	Sn-Glycero-3-phosphocholine	C8H20NO6P	0	C_e
LalaDgluMdapDala_e	L-alanine-D-glutamate-meso-2,6-diaminoheptanedioate-D-alanine	C18H30N5O9	-1	C_e
glcur_e	D-Glucuronate	C6H9O7	-1	C_e
dgsn_e	Deoxyguanosine	C10H13N5O4	0	C_e
udcpdp_e	Undecaprenyl diphosphate	C55H90O7P2	0	C_e
alaala_e	D-Alanyl-D-alanine	C6H12N2O3	0	C_e
doxrbcn_e	Doxorubicin	C27H30NO11	1	C_e
ala__D_e	D-Alanine	C3H7NO2	0	C_e
pyovd_kt_e	Pyoverdine P putida specific	C27H48N10O15	0	C_e
galt_e	Galactitol	C6H14O6	0	C_e
icit_e	Isocitrate	C6H5O7	-3	C_e
amp_e	AMP C10H12N5O7P	C10H12N5O7P	-2	C_e
met__L_e	L-Methionine	C5H11NO2S	0	C_e
rmn_e	L-Rhamnose	C6H12O5	0	C_e
ocdcea_e	Octadecenoate (n-C18:1)	C18H33O2	-1	C_e
dxyl_e	1-deoxy-D-xylulose	C5H10O4	0	C_e
pydxn_e	Pyridoxine	C8H11NO3	0	C_e
ile__L_e	L-Isoleucine	C6H13NO2	0	C_e
man_e	D-Mannose	C6H12O6	0	C_e
fe3dcit_e	Fe(III)dicitrate	C12FeH10O14	0	C_e
glc__D_e	D-Glucose	C6H12O6	0	C_e
xtsn_e	Xanthosine	C10H12N4O6	0	C_e
octa_e	Octanoate (n-C8:0)	C8H15O2	-1	C_e
co2_e	CO2 CO2	CO2	0	C_e
fuc__L_e	L-Fucose	C6H12O5	0	C_e

duri_e	Deoxyuridine	C9H12N2O5	0	C_e
glyclt_e	Glycolate C2H3O3	C2H3O3	-1	C_e
asn__L_e	L-Asparagine	C4H8N2O3	0	C_e
glyc_e	Glycerol	C3H8O3	0	C_e
hxa_e	Hexanoate (n-C6:0)	C6H11O2	-1	C_e
pacald_e	Phenylacetaldehyde	C8H8O	0	C_e
acmana_e	N-Acetyl-D-mannosamine	C8H15NO6	0	C_e
2ameph_e	2-Aminoethylphosphonate	C2H8NO3P	0	C_e

Table S12- Properties of metabolites used in this study; we performed simulations on each GEM by letting the exchange reactions of amino acids to -1 mmol/g_{DW}/h and changing the carbon source including starch, maltose, cellobiose, and glucose [-5 mmol/g_{DW}/h]. we performed *p*FBA and recorded all nonzero exchange reactions from all simulations for the GEMs (70 metabolites).

Name	Diffusion coefficient (10^{-9} m^2/s)	Molecular Weight (g/mol)	Molecular Weight (g/mol)
4-Aminobutanoate	0.76	103.12	Estimated based on GABA from Diffusion of GABA in brain tissue (Nicholson, 1985)
Acetate	1.2	59.04	Diffusion coefficients of acetate ions in aqueous solutions (Barry & Diamond, 1984)
Acetaldehyde	1.4	44.05	Estimated based on formaldehyde (similar small aldehyde)
Adenine	0.45	135.13	Estimated from molecular weight using Wilke-Chang equation
Adenosine	0.5	267.24	Diffusion coefficients of nucleosides in water (Cussler, 1997)
L-Alanine	0.9	89.09	Diffusion coefficients of amino acids (Robinson & Stokes, 1968)
Allantoin	0.57	158.12	Estimated from uric acid (similar compound)
L-Arabinose	0.6	150.13	Diffusion coefficients of monosaccharides (Robinson & Stokes, 1968)
Arbutin C12H16O7	0.45	272.25	Estimated from glucose derivatives (similar size and structure)
L-Arginine	0.75	174.2	Diffusion coefficients of amino acids (Robinson & Stokes, 1968)
L-Ascorbate	0.6	176.12	Diffusion coefficient of ascorbic acid (Zheng & Wang, 2001)
L-Asparagine	0.8	132.12	Diffusion coefficients of amino acids (Robinson & Stokes, 1968)
L-Aspartate	0.76	133.1	Diffusion coefficients of amino acids (Robinson & Stokes, 1968)
Biotin	0.38	244.31	Estimated from molecular weight using Wilke-Chang equation
Butyrate (n-C4:0)	1	88.11	Diffusion coefficients of short-chain fatty acids (Barry & Diamond, 1984)
Benzoate	0.85	122.12	Diffusion coefficients of aromatic compounds (Robinson & Stokes, 1968)
L-Carnosine	0.54	226.23	Estimated from similar dipeptides
Cellobiose	0.43	342.3	Diffusion coefficients of disaccharides (Robinson & Stokes, 1968)
Cys Gly C5H10N2O3S	0.7	162.21	Estimated based on glycine and cysteine
Choline C5H14NO	0.7	104.17	Diffusion coefficients of quaternary ammonium compounds (Gamble, 1984)
Choline sulfate	0.65	185.2	Estimated from choline with sulfate adjustment
Citrate	0.55	192.13	Diffusion coefficient of citrate ions (Barry & Diamond, 1984)
CO2	1.9	44.01	Diffusion of gases in water (Wilke & Chang, 1955)
L-Cysteine	0.83	121.16	Diffusion coefficients of amino acids (Robinson & Stokes, 1968)
Dextrin C12H20O10	0.1	324.32	Estimated from dextrans diffusion characteristics (Berth et al 2002)
Folate	0.35	441.4	Estimated from molecular weight using Wilke-Chang equation
D-Fructose	0.6	180.16	Diffusion coefficients of monosaccharides (Robinson & Stokes, 1968)
D-Glucose	0.67	180.16	Diffusion coefficients of monosaccharides (Robinson & Stokes, 1968)

L-Glutamine	0.75	146.15	Diffusion coefficients of amino acids (Robinson & Stokes, 1968)
L-Glutamate	0.76	147.13	Diffusion coefficients of amino acids (Robinson & Stokes, 1968)
Glycine	1	75.07	Diffusion coefficients of amino acids (Robinson & Stokes, 1968)
Hydrogen	4.5	2.02	Diffusion of gases in water (Wilke & Chang, 1955)
L-Histidine	0.6	155.16	Diffusion coefficients of amino acids (Robinson & Stokes, 1968)
L-Isoleucine	0.75	131.18	Diffusion coefficients of amino acids (Robinson & Stokes, 1968)
Indole	0.5	117.15	Estimated from molecular weight and structure similarity
Lactose C12H22O11	0.41	342.3	Diffusion coefficients of disaccharides (Robinson & Stokes, 1968)
L-Leucine	0.75	131.18	Diffusion coefficients of amino acids (Robinson & Stokes, 1968)
L-Lysine	0.6	146.19	Diffusion coefficients of amino acids (Robinson & Stokes, 1968)
M-Xylene	1.24	106.17	Diffusion coefficients of aromatic hydrocarbons (Robinson & Stokes, 1968)
L-Malate	0.53	134.09	Diffusion coefficients of dicarboxylate ions (Barry & Diamond, 1984)
Maltose C12H22O11	0.43	342.3	Diffusion coefficients of disaccharides (Robinson & Stokes, 1968)
L-Methionine	0.65	149.21	Diffusion coefficients of amino acids (Robinson & Stokes, 1968)
O2 O2	2.1	32	Diffusion of gases in water (Wilke & Chang, 1955)
L-Phenylalanine	0.55	165.19	Diffusion coefficients of amino acids (Robinson & Stokes, 1968)
Protoheme C34H30Feds7N4O4	0.32	616.49	Estimated from Stokes-Einstein equation (k_B=1.38e-23J/K; T = 310 K; η = 0.69e-3 Pa·s; r = 1e-9 m)
Propionate (n-C3:0)	1.15	74.08	Diffusion coefficients of short-chain fatty acids (Barry & Diamond, 1984)
Propanoyl phosphate	0.9	122.08	Estimated based on molecular size and charge
L-Proline	0.68	115.13	Diffusion coefficients of amino acids (Robinson & Stokes, 1968)
D-Ribose	0.6	150.13	Diffusion coefficients of monosaccharides (Robinson & Stokes, 1968)
Riboflavin C17H20N4O6	0.32	376.37	Diffusion coefficients of vitamins in water (Lee, 1977)
L-Rhamnose	0.56	164.16	Estimated from similar monosaccharides
D-Sorbitol	0.52	182.17	Diffusion coefficients of sugar alcohols (Robinson & Stokes, 1968)
L-Serine	0.9	105.09	Diffusion coefficients of amino acids (Robinson & Stokes, 1968)
Starch C12H20O10	0.1	324.32	Estimated from dextrans diffusion characteristics (Berth et al 2002)
Succinate	0.8	118.09	Diffusion coefficients of dicarboxylate ions (Barry & Diamond, 1984)
Sucrose C12H22O11	0.45	342.3	Diffusion coefficients of disaccharides (Robinson & Stokes, 1968)
Thiamin	0.36	265.35	Diffusion coefficients of vitamins in water (Lee, 1977)
L-Threonine	0.8	119.12	Diffusion coefficients of amino acids (Robinson & Stokes, 1968)
Thymine C5H6N2O2	0.65	126.11	Estimated from similar nucleobases
Toluene	1.4	92.14	Diffusion coefficients of aromatic hydrocarbons (Robinson & Stokes, 1968)

L-Tryptophan	0.53	204.23	Diffusion coefficients of amino acids (Robinson & Stokes, 1968)
L-Tyrosine	0.5	181.19	Diffusion coefficients of amino acids (Robinson & Stokes, 1968)
Uracil	0.72	112.09	Estimated from similar nucleobases
Uridine	0.65	244.2	Diffusion coefficients of nucleosides in water (Cussler, 1997)
L-Valine	0.85	117.15	Diffusion coefficients of amino acids (Robinson & Stokes, 1968)
D-Xylose	0.63	150.13	Diffusion coefficients of monosaccharides (Robinson & Stokes, 1968)
Putrescine	1	88.15	Estimated from similar polyamines
Ornithine	0.8	132.17	Diffusion coefficients of amino acids (Robinson & Stokes, 1968)
D-Lactate	0.9	90.08	Diffusion coefficients of organic acids (Robinson & Stokes, 1968)
Formate	1.18	46.03	Diffusion coefficients of small organic acids (Barry & Diamond, 1984)

Table S13- Genes with notable flux shifts across the domain; Results highlighting *E. coli* enzymes with notable flux shifts across the domain. All the transport reactions were excluded from this figure (fluxes reported in mmol//g_{DW}/h).

Rxn ID	Gene	Reaction Name	Reaction Stoichiometry
ACALD	[NP_414885_1, NP_415757_1, NP_416950_1]	Acetaldehyde dehydrogenase (acetylating)	acald_c + coa_c + nad_c <=> accoa_c + h_c + nadh_c
ACKr	[NP_417585_2, NP_416363_1, NP_416799_1]	Acetate kinase	ac_c + atp_c <=> actp_c + adp_c
ACLS	[NP_414620_1, NP_418127_1, NP_418217_1, YP_025294_2, NP_418126_1]	Acetolactate synthase	h_c + 2.0 pyr_c --> alac__S_c + co2_c
ADK1	[NP_415007_1]	Adenylate kinase	amp_c + atp_c <=> 2.0 adp_c
ADK3	[NP_415007_1]	Adentylate kinase (GTP)	amp_c + gtp_c <=> adp_c + gdp_c
AKGDH	[NP_415255_1, NP_414658_1, NP_415254_1]	2-Oxogluterate dehydrogenase	akg_c + coa_c + nad_c --> co2_c + nadh_c + succoa_c
ALAR	[NP_418477_1, NP_415708_1, NP_417426_1]	Alanine racemase	ala__L_c <=> ala__D_c
ALCD2x	[NP_415757_1, NP_414890_1, NP_415995_4, NP_416948_4]	Alcohol dehydrogenase (ethanol)	etoh_c + nad_c <=> acald_c + h_c + nadh_c
ASAD	[NP_417891_1, NP_416822_1]	Aspartate-semialdehyde dehydrogenase	aspsa_c + nadp_c + pi_c <=> 4pasp_c + h_c + nadph_c
ASPK	[NP_414543_1, NP_418448_1, NP_418375_1]	Aspartate kinase	asp__L_c + atp_c <=> 4pasp_c + adp_c
ASPTA	[NP_415448_1, NP_418478_1, NP_415133_1]	Aspartate transaminase	akg_c + asp__L_c <=> glu__L_c + oaa_c
ASR	[NP_416990_1, NP_417960_1, NP_415582_1]	Arsenate reductase	aso4_c + 2.0 gthrd_c + 2.0 h_c --> aso3_c + gthox_c + h2o_c
ATHRDHr	[NP_416057_1]	L-allo-threonine dehydrogenase	athr__L_c + nadp_c <=> 2aobut_c + h_c + nadph_c
CS	[NP_415248_1]	Citrate synthase	accoa_c + h2o_c + oaa_c --> cit_c + coa_c + h_c
CYSS	[NP_416909_1, NP_416916_1]	Cysteine synthase	acser_c + h2s_c --> ac_c + cys__L_c + h_c
DHAD1	[YP_026248_1]	Dihydroxy-acid dehydratase (2,3-dihydroxy-3-methylbutanoate)	23dhmb_c --> 3mob_c + h2o_c
DHAK	[NP_418715_2]	Dihydroxyacetone kinase	atp_c + dha_c --> adp_c + dhap_c + h_c
DHAPT	[NP_415718_6, NP_415716_4, NP_416910_1, NP_416911_1, NP_415717_1, YP_026278_1]	Dihydroxyacetone phosphotransferase	dha_c + pep_c --> dhap_c + pyr_c
EDA	[NP_416364_1]	2-dehydro-3-deoxy-phosphogluconate aldolase	2ddg6p_c --> g3p_c + pyr_c
EDD	[NP_416365_1]	6-phosphogluconate dehydratase	6pgc_c --> 2ddg6p_c + h2o_c
F6PA	[NP_418381_1, NP_415346_4]	Fructose 6-phosphate aldolase	f6p_c <=> dha_c + g3p_c
FBA	[NP_417400_1, NP_416287_1, NP_416600_4]	Fructose-bisphosphate aldolase	fdp_c <=> dhap_c + g3p_c
FE3Ri	[NP_418286_1]	Fe(III) reduction	fadh2_c + 2.0 fe3_c --> fad_c + 2.0 fe2_c + 2.0 h_c
FESR	[NP_418630_1]	Iron-sulfur cluster repair	3fe4s_c + fe2_c --> 4fe4s_c
FTHFD	[NP_415748_1]	Formyltetrahydrofolate deformylase	10fthf_c + h2o_c --> for_c + h_c + thf_c
G6PDH2r	[NP_416366_1]	Glucose 6-phosphate dehydrogenase	g6p_c + nadp_c <=> 6pgl_c + h_c + nadph_c
GAPD	[NP_416293_1]	Glyceraldehyde-3-phosphate dehydrogenase	g3p_c + nad_c + pi_c <=> 13dpg_c + h_c + nadh_c
GK1	[NP_418105_1]	Guanylate kinase (GMP:ATP)	atp_c + gmp_c <=> adp_c + gdp_c
GND	[NP_416533_1]	Phosphogluconate dehydrogenase	6pgc_c + nadp_c --> co2_c + nadph_c + ru5p__D_c
GTHOr	[NP_417957_1]	Glutathione oxidoreductase	gthox_c + h_c + nadph_c <=> 2.0 gthrd_c + nadp_c

HEX1	[NP_416111_1, NP_415202_1, NP_416889_1]	Hexokinase (D-glucose:ATP)	atp_c + glc__D_c --> adp_c + g6p_c + h_c
HSDy	[NP_414543_1, NP_418375_1]	Homoserine dehydrogenase (NADPH)	hom__L_c + nadp_c <=> aspsa_c + h_c + nadph_c
HSK	[NP_414544_1]	Homoserine kinase	atp_c + hom__L_c --> adp_c + h_c + phom_c
IPMD	[NP_416314_1, NP_414615_4]	3-isopropylmalate dehydrogenase	3c2hmp_c + nad_c --> 3c4mop_c + h_c + nadh_c
IPPMIa	[NP_414613_1, NP_414614_1]	3-isopropylmalate dehydratase	3c2hmp_c <=> 2ippm_c + h2o_c
IPPMIb	[NP_414613_1, NP_414614_1]	2-isopropylmalate hydratase	2ippm_c + h2o_c <=> 3c3hmp_c
IPPS	[NP_414616_1]	2-isopropylmalate synthase	3mob_c + accoa_c + h2o_c --> 3c3hmp_c + coa_c + h_c
KARA1	[NP_418222_1]	Ketol-acid reductoisomerase (2,3-dihydroxy-3-methylbutanoate)	23dhmb_c + nadp_c <=> alac__S_c + h_c + nadph_c
LEUTAi	[YP_026247_1, NP_418478_1]	Leucine transaminase (irreversible)	4mop_c + glu__L_c --> akg_c + leu__L_c
MDH	[NP_417703_1, NP_417241_1]	Malate dehydrogenase	mal__L_c + nad_c <=> h_c + nadh_c + oaa_c
MTHFC	[NP_415062_1]	Methenyltetrahydrofolate cyclohydrolase	h2o_c + methf_c <=> 10fthf_c + h_c
NDPK1	[NP_417013_1, NP_415007_1]	Nucleoside-diphosphate kinase (ATP:GDP)	atp_c + gdp_c <=> adp_c + gtp_c
NODOy	[NP_417047_1]	Nitric oxide dioxygenase	nadph_c + 2.0 no_c + 2.0 o2_c --> h_c + nadp_c + 2.0 no3_c
NTRIR2x	[NP_417824_1, NP_417825_1]	Nitrite Reductase (NADH)	5.0 h_c + 3.0 nadh_c + no2_c --> 2.0 h2o_c + 3.0 nad_c + nh4_c
OMCDC	[NP_414615_4, spontaneous]	2-Oxo-4-methyl-3-carboxypentanoate decarboxylation	3c4mop_c + h_c --> 4mop_c + co2_c
PFK	[NP_416237_3, NP_418351_1]	Phosphofructokinase	atp_c + f6p_c --> adp_c + fdp_c + h_c
PGI	[NP_418449_1]	Glucose-6-phosphate isomerase	g6p_c <=> f6p_c
PGK	[NP_417401_1]	Phosphoglycerate kinase	3pg_c + atp_c <=> 13dpg_c + adp_c
PGL	[NP_415288_1]	6-phosphogluconolactonase	6pgl_c + h2o_c --> 6pgc_c + h_c
PTAr	[NP_416800_1, NP_416953_1]	Phosphotransacetylase	accoa_c + pi_c <=> actp_c + coa_c
RPE	[NP_418721_1, NP_417845_1]	Ribulose 5-phosphate 3-epimerase	ru5p__D_c <=> xu5p__D_c
RPI	[NP_417389_1, NP_418514_1]	Ribose-5-phosphate isomerase	r5p_c <=> ru5p__D_c
SLCYSS	[NP_416916_1]	O-acetyl-L-serine sulfhydrylase	acser_c + tsul_c --> ac_c + scys__L_c
SUCOAS	[NP_415257_1, NP_415256_1]	Succinyl-CoA synthetase (ADP-forming)	atp_c + coa_c + succ_c <=> adp_c + pi_c + succoa_c
TALA	[NP_416959_1, NP_414549_1]	Transaldolase	g3p_c + s7p_c <=> e4p_c + f6p_c
THRA	[NP_417046_1, NP_415391_1]	Threonine aldolase	thr__L_c --> acald_c + gly_c
THRA2	[NP_417046_1, NP_415391_1]	L-allo-Threonine Aldolase	athr__L_c --> acald_c + gly_c
THRS	[NP_414545_1]	Threonine synthase	h2o_c + phom_c --> pi_c + thr__L_c
TKT1	[NP_416960_1, YP_026188_1]	Transketolase	r5p_c + xu5p__D_c <=> g3p_c + s7p_c
TKT2	[NP_416960_1, YP_026188_1]	Transketolase	e4p_c + xu5p__D_c <=> f6p_c + g3p_c
XYLI2	[NP_418022_1]	Xylose isomerase	glc__D_c <=> fru_c

Table S14- Size of girds used for different experiments.

**Scenario**	**length (µm)**	**Width (µm)**	**Lattice site (µm)**	**Biofilm length (µm)**
Scenario 1	400	120	10	100
Scenario 2	400	120	10	100
High protein-diet on overweight individuals	800	100	10	80
